# Amyloid precursor protein selective gamma-secretase inhibitors for treatment of Alzheimer's disease

**DOI:** 10.1186/alzrt60

**Published:** 2010-12-29

**Authors:** Guriqbal S Basi, Susanna Hemphill, Elizabeth F Brigham, Anna Liao, Danielle L Aubele, Jeanne Baker, Robin Barbour, Michael Bova, Xiao-Hua Chen, Michael S Dappen, Tovah Eichenbaum, Erich Goldbach, Jon Hawkinson, Rose Lawler-Herbold, Kang Hu, Terence Hui, Jacek J Jagodzinski, Pamela S Keim, Dora Kholodenko, Lee H Latimer, Mike Lee, Jennifer Marugg, Matthew N Mattson, Scott McCauley, James L Miller, Ruth Motter, Linda Mutter, Martin L Neitzel, Huifang Ni, Lan Nguyen, Kevin Quinn, Lany Ruslim, Christopher M Semko, Paul Shapiro, Jenifer Smith, Ferdie Soriano, Balazs Szoke, Kevin Tanaka, Pearl Tang, John A Tucker, Xiacong Michael Ye, Mei Yu, Jing Wu, Ying-zi Xu, Albert W Garofalo, John Michael Sauer, Andrei W Konradi, Daniel Ness, George Shopp, Michael A Pleiss, Stephen B Freedman, Dale Schenk

**Affiliations:** 1 Elan Pharmaceuticals, Inc. 180 Oyster Point Blvd., S. San Francisco, CA 94080, USA; 2 Neotope Biosciences Inc., 650 Gateway Blvd., S San Francisco, CA 94080, USA

## Abstract

**Introduction:**

Inhibition of gamma-secretase presents a direct target for lowering Aβ production in the brain as a therapy for Alzheimer's disease (AD). However, gamma-secretase is known to process multiple substrates in addition to amyloid precursor protein (APP), most notably Notch, which has limited clinical development of inhibitors targeting this enzyme. It has been postulated that APP substrate selective inhibitors of gamma-secretase would be preferable to non-selective inhibitors from a safety perspective for AD therapy.

**Methods:**

*In vitro *assays monitoring inhibitor potencies at APP γ-site cleavage (equivalent to Aβ40), and Notch ε-site cleavage, in conjunction with a single cell assay to simultaneously monitor selectivity for inhibition of Aβ production vs. Notch signaling were developed to discover APP selective gamma-secretase inhibitors. *In vivo *efficacy for acute reduction of brain Aβ was determined in the PDAPP transgene model of AD, as well as in wild-type FVB strain mice. *In vivo *selectivity was determined following seven days x twice per day (b.i.d.) treatment with 15 mg/kg/dose to 1,000 mg/kg/dose ELN475516, and monitoring brain Aβ reduction vs. Notch signaling endpoints in periphery.

**Results:**

The APP selective gamma-secretase inhibitors ELN318463 and ELN475516 reported here behave as classic gamma-secretase inhibitors, demonstrate 75- to 120-fold selectivity for inhibiting Aβ production compared with Notch signaling in cells, and displace an active site directed inhibitor at very high concentrations only in the presence of substrate. ELN318463 demonstrated discordant efficacy for reduction of brain Aβ in the PDAPP compared with wild-type FVB, not observed with ELN475516. Improved *in vivo *safety of ELN475516 was demonstrated in the 7d repeat dose study in wild-type mice, where a 33% reduction of brain Aβ was observed in mice terminated three hours post last dose at the lowest dose of inhibitor tested. No overt in-life or post-mortem indications of systemic toxicity, nor RNA and histological end-points indicative of toxicity attributable to inhibition of Notch signaling were observed at any dose tested.

**Conclusions:**

The discordant *in vivo *activity of ELN318463 suggests that the potency of gamma-secretase inhibitors in AD transgenic mice should be corroborated in wild-type mice. The discovery of ELN475516 demonstrates that it is possible to develop APP selective gamma-secretase inhibitors with potential for treatment for AD.

## Introduction

The principal pathological features of Alzheimer's disease (AD), comprised of neurofibrilary tangles and amyloid plaques, are posited by the amyloid cascade hypothesis [1-3] to be pivotal in the clinical manifestations (impaired memory and cognition, dementia) of the disease. Current marketed therapies for AD offer palliative cognitive benefits with little to no impact on the underlying pathology, or on long-term disease progression. Effective treatments for AD that address the underlying disease represent a major unmet medical need.

Immunotherapy targeting Aβ has been demonstrated to modify amyloid [4,5] as well as tau related endpoints [6,7] of AD pathology in pre-clinical models, as well as human clinical trials, and is currently in advanced clinical trials for potential treatment of mild to moderate AD [8,9]. Orally bioavailable small molecule therapeutics offer the desirable attributes of convenient administration combined with in-home use for chronic therapy of AD, and as such, are anticipated to fill an unmet need in the emerging landscape of next generation AD therapeutics.

Pharmacological inhibition of gamma-secretase *in vivo *is a well-documented small molecule target for lowering brain, CSF, and plasma Aβ peptide [10-18], and impacting AD pathology [14,19-22]. Gamma-secretase inhibitors (GSIs) have also shown benefits on presumed correlates of memory in AD transgene models under acute [23], as well as, chronic treatment paradigms [24]. Consequently, gamma-secretase has been the target of ongoing medicinal chemistry efforts to discover therapeutics for treatment of AD [25-27]. However, inhibition of Notch processing by non-selective GSI's manifests in dysregulated cellular homeostasis and non-target organ side effects, for example, goblet cell hyperplasia in the gastrointestinal tract [28-30], that translate to clinical observations [31-33], and present challenges for clinical development of first generation GSI's [34]. Support for the observation that pharmacological effects of GSI's on cellular homeostasis in the gastro-intestinal tract are due to dysregulation of Notch pathway derives from observations with genetic knock-out [35-38] as well as gain of function mouse models [39] of Notch pathway genes.

Approaches to managing gastro-intestinal side effects of first generation GSIs via intermittent dosing [40,41] or glucocorticoid therapy [42] have been demonstrated in pre-clinical models. Additional efforts targeting gamma-secretase for AD therapy have been influenced by gamma-secretase cleavage site modulating properties of certain NSAIDS [43-45], and APP substrate selective/Notch sparing GSIs (this report, [46-48]) as a means toward mitigating inhibition of Notch signaling. Clinical development of the most advanced NSAID based gamma-secretase modulator, tarenflurbil, was discontinued due to lack of efficacy in P3 clinical trial [49,50], however, second generation candidates are progressing through both clinical [51] as well as preclinical stages of development [52-55]. Additionally, a nucleotide binding site on presenilin has also been reported to inhibit Aβ while sparing Notch [56-58], and offers another avenue under investigation for the next generation of gamma-secretase inhibitors.

The pharmacological and genetic evidence cited above validate gamma-secretase as a target for lowering Aβ production as well as non-target organ side effects due to inhibition of Notch signaling. Together, the observations support the hypothesis that APP selective gamma-secretase inhibitors offer one approach toward potentially safer gamma secretase targeted therapeutics for AD. Toward that end, we report here the discovery of novel APP selective inhibitors of gamma-secretase discovered from a high throughput screen of a chemical library enabled by novel assays for comparing APP and Notch cleavage by gamma-secretase. We confirmed that the improved *in vitro *selectivity of our lead compound, ELN475516 translates into improved *in vivo *safety in a mouse model that is sensitive to histological and molecular end-points associated with inhibition of Notch signaling.

## Materials and methods

### Compounds

ELN46719 is the 2-hydroxy-valeric acid amide analog of LY411575 (where LY411575 is the 3,5-difluoro-mandelic acid amide) (US Patent No 6,541,466). ELN318463 was described by Zhao *et al. *[59], and ELN475516 has been described as compound 11a by Mattson *et al. *[60].

### Antibodies and substrates

Notch intracellular domain (NICD) neo-epitope monoclonal antibody (mAb) 9F3 was generated by immunizing mice with VLLSRGGC (corresponding to amino-terminus of human NICD, residues 1755 to 1759 in full length Notch) coupled to maleimide activated sheep anti-mouse IgG. Spleenocytes from the highest antibody titer mouse were fused with mouse myeloma cells. Hybridomas were screened against VLLSRGGC and counter screened against GCGVLLSR. Monoclonal antibody 9F3 only recognized VLLSRGGC peptide. The specificity of 9F3 for recognition of the amino-terminal neoepitope of NICD product, revealed from *in vitro *digestion of NotchΔE substrate by gamma-secretase enzyme, but not the uncleaved NotchΔE substrate, was confirmed by Western blot assay. The sequence of this epitope is conserved between human and mouse Notch1.

*E. coli *expressed MBP-APPc125sw fusion protein substrate [61], was purified by amylose affinity chromatography per manufacturer's protocol (New England Biolabs, Ipswich, Massachusetts, USA) and stored at -40°C as 2 mg/mL stock in 3 M guanidine-HCl, 1% Triton-X100, 20 mM Tris, (pH 7.5) until use.

*NotchΔEΔC substrate: *A Notch fusion protein was constructed so as to be analogous to the APP C99 fragment as described [62]. Specifically, the Notch fusion protein is comprised of 99 amino-acids flanking the trans-membrane domain of mouse Notch1, accession (Genbank: Z11886). The amino-terminus of the fusion protein begins at residue #1711 of mouse Notch (numbered from initiator methionine residue), and extends carboxy-terminal to residue #1809, followed by in-frame fusion with HA epitope tag and six histidine residues prior to the stop codon. The two carboxy-terminal epitope tags are separated by an "LE" di-peptide spacer. The epitope tags were incorporated to facilitate ELISA detection of the NICD cleavage product using anti-HA antibody, and purification of the fusion protein substrate from *E.coli *lysates using nickel column affinity chromatography, respectively. Following overnight expression of the fusion protein in *E.coli*, the substrate is purified on a nickel-sepharose column from lysates. Purity of the 16 kD Notch fusion protein substrate eluted from the nickel-affinity column is confirmed by SDS-PAGE. Fractions containing the desired protein were pooled, adjusted to 0.5 to 0.6 mg/mL final concentration in 3M Guanidine-HCl, 0.1% Triton X-100 with 20 mM DTT, and stored at -80°C in aliquots until use in the enzyme reaction. This protein is not stable over long periods, and will aggregate even in the above storage buffer at -80°C. Hence, a new batch is prepared every three months to ensure assay quality. The substrate is desalted over a NAP-25 column immediately prior to use, protein concentration is determined by BCA assay (Pierce, Rockford, Illinois, USA), and diluted 20X for incubation with enzyme.

*NICD ELISA standard: *A human NICD standard was produced as a fusion protein from a construct in the bacterial expression vector pCal-kc (Invitrogen, Carlsbad, California, USA). The expression construct was modified to encode an initiating methionine followed by an enterokinase cleavage at the N-terminus, fused in frame to the gamma-secretase product NICD (Met-**EK**-NICD, where NICD comprises residues 1755 to 1875 of human Notch1, numbering from the initiator methionine), an 8 residue spacer comprising the sequence PAAAAAA, and an HA epitope tag fused in frame with vector derived kemptide peptide, thrombin cleavage site and carboxy-terminal calmodulin binding peptide. The fusion protein was produced in *E.coli*, affinity purified using a calmodulin affinity resin and assayed for protein concentration. The protein was then cleaved with enterokinase in order to produce the free N-terminus of the NICD product. The enterokinase cleaved protein was separated by SDS-PAGE on a 12% Tris-glycine gel. Enterokinase cleavage products are detected by Western blotting using an antibody against the HA-tag. Densitometry was used to measure the efficiency of the enterokinase cleavage. The estimated concentration of enterokinase cleaved NICD standard is calculated (% of NICD/total HA-reactive protein). The standard was stored at -40°C. On the day of each assay, an aliquot of the NICD standard is serially diluted 1:1 in casein diluent to generate a standard curve with 0 to 200 ng/mL NICD.

### Enzyme preparation and *in vitro *assays for recombinant APP and Notch substrate cleavage

An enriched detergent solubilized gamma-secretase enzyme preparation from IMR-32 neuroblastoma cells was obtained by membrane fractionation of crude homogenates using ultracentrifugation, followed by lectin affinity chromatography. Briefly, a 100,000 g membrane pellet of post nuclear superantant from IMR32 frozen cell pellets was homogenized in buffer (250 mM sucrose, 10 mM HEPES (pH 7.5), 1 mM MgCl_2_, 0.2 mM CaCl_2 _+ protease inhibitors, 5 mL/g cell pellet), resuspended in an equal volume of hypotonic buffer (homogenization buffer minus sucrose), subjected to freeze thaw (dry-ice, 37°C water bath), and re-centrifuged at 100,000 x g for 20 minutes at 4°C. The membrane pellet was snap frozen on dry ice, and stored at -80°C until extraction. Thawed membranes were washed in 1 M carbonate buffer (1 M Na_2_CO_3_, 1 mM MgCl_2_, 0.2 mM CaCl_2_) at 4°C for 15 minutes, centrifuged 100,000 X g as explained above, and resuspended in hypotonic wash buffer. Washed membrane pellets were resuspended in 0.5X final volume of solubilization buffer (10 mM CHES pH 9.5, 50 mM NaCl, 1 mM MgCl_2_, 0.2 mM CaCl_2 _+ protease inhibitors, minus pepstatin) and extracted in detergent containing 1.49 mg/mL BigCHAP (ICN), diluted fresh 1:10 from 50X stock (149 mg/mL in H_2_O), at 4°C for 1.5 h. Detergent extracts were centrifuged at 100,000 x g as explained above, and supernatant containing solubilized gamma-secretase aspirated for WGA chromatography. Peak eluate fractions were combined, and stored in 10 mM Hepes, pH 7.5, 1 M N-acetyl-D-glucosamine, 1 mM MgCl_2_, 0.2 mM CaCl_2_, 0.6 mg/mL TypeV brain extract (Sigma, St. Louis, Missouri, USA), 5.6 mg/mL BigCHAP.

### *In vitro *assay for cleavage of recombinant fusion protein substrates by partially purified gamma-secretase enzyme from IMR-32 cells

The *in vitro *gamma-secretase assay was designed to measure the specific proteolytic cleavage of an APP substrate (MBP-APPc125Sw fusion protein) at the Aβ40 site, or cleavage of Notch fusion protein at the Notch ε-site (corresponding to N-terminus of NICD). The substrates were incubated in separate reactions, in triplicate, with an enriched preparation of the enzyme from IMR-32 cells, and the amount of specific product formed was measured using a sandwich ELISA comprised of an Aβ40-specific capture antibody, 2G3, and a biotinylated Aβ17-28 reporter antibody, 6H9 for APP substrate, or Notch ε-site specific antibody 9F3, specific for the amino-terminus of the NICD (beginning at V1755 of human Notch protein), as capture and anti-HA epitope tag antibody as the reporter for Notch substrate. Incubation of triplicate reactions in the presence of a range of inhibitor concentrations enabled determination of a dose-response for enzyme inhibition by the test compound and calculation of an IC_50 _value. Data analysis was performed using XLfit software package (IDBS Software Inc. Alalmeda, California, USA).

Recombinant APP or Notch fusion protein substrates (purified as described above) were incubated in reaction buffer (50 mM MES, pH 6.0, 400 μg/mL Type-V phospholipid, 5.6 mg/mL BigCHAP, 4 mM DTT, 0.02% TX-100) at 20 μg/mL or 30 ug/mL, respectively, with approximately 0.4 mg/mL enzyme preparation (1:100 to 1:250 dilution, depending upon batch to batch variation of specific activity) for 2 h at 37°C in the presence of protease inhibitors (1 mM 1,10-phenanthroline, 5 ug/mL E64, and 5 ug/mL leupeptin) in the presence of a concentration range of test inhibitor compounds. Reactions were quenched with 0.1% SDS for 10 minutes at room temperature, followed by addition of equal volume of specimen diluent (1.5 mM NaH_2_PO_4_^.^H_2_O, 8 mM Na_2_HPO_4_^.^7H_2_O, 8 mM NaN_3_, 150 mM NaCl, 0.05% (volume/volume) Triton X-405 0.6% (w/v) BSA, (globulin free).

### ELISA detection of Aβ40 cleavage product

The diluted enzyme reaction mixture was transferred to antibody 2G3-coated Immulon plates (specific for Aβ40 carboxy-terminal neo-epitope) and incubated overnight along with Aβ1 to 40 standards (32 to 2,000 pg/mL in Specimen diluent). The ELISA was developed the following day by sequential one hour incubations with biotinylated 6H9 reporter antibody (Elan, Aβ 17-28 specific, 0.25 μg/mL) for one hour at room temperature, and Streptavidin-Alkaline Phosphatase Conjugate (Roche Molecular Biochemicals, Catalog No. 1089 161, Indianapolis, Indiana, USA), 1:1,000 in specimen diluent, one hour room temperature. The ELISA was developed using fluorescent alkaline phosphatase substrate, and the plate is read at 360 nm excitation/460 nm emission, gain approximately 50, in a CytoFluor 4000 (Applied Biosystems, Foster City, California, USA). The plates were washed 3X each with Tris-buffered saline/0.05% Tween-20 between incubations with reporter antibody, detecting antibody, and fluorescent substrate.

### ELISA detection of NICD product

Quenched *in vitro *gamma/Notch reactions were diluted 1:1 with casein diluent buffer containing 500 mM NaCl, 0.02% TritonX-100 and plated onto Notch ε-cleavage site specific neoepitope mAb 9F3 (specific for the amino-terminus of NICD, beginning at V1755 of human Notch) coated immulon plates, for capture of the NICD reaction product overnight at 4°C. NICD standards, prepared as described above, were serially diluted 2X over a concentration range from a starting concentration of 50 ng/mL for establishing a standard curve. The NICD reaction product and standards captured on the plate are detected with biotinylated-HA antibody (1 μg/mL final, Roche Cat# 1666851), followed by alkaline phosphatase conjugated streptavidin (diluted 1,000X from stock, Roche Cat# 1089161). The plate was washed between each incubation step with Tris-buffered saline containing 0.1% Tween-20. The alkaline phosphatase reaction product is detected by incubation with 100 μl/well Fluorescent Substrate A for 15 minutes at room temperature, and detected using Cytofluor plate reader at 360 nm excitation, 460 nm emission.

### Dual assay for simultaneous inhibition of Aβ and Notch signaling in cells

In order to test the potency and selectivity of gamma-secretase inhibitors against two substrates (APP and Notch) simultaneously, we developed a dual-assay CHO cell line stably expressing APPSw, a Notch substrate lacking the ecto-domain, and a Notch responsive Luciferase reporter gene. As a first step, CHO cells stably over-expressing the APPsw were established. These cells secrete high levels of Aβ peptide into the conditioned media from endogenous β- and γ-secretase enzyme activity. A Notch intracellular domain responsive reporter gene, and the constitutive gamma-secretase Notch substrate, NotchΔE, were stably introduced into CHOsw cells in a two-step process. CHOsw cells were first stably transfected to express a NICD responsive luciferase reporter gene construct (pGL2-CBF-Luc) [63]. Numerous stable clones (SCH clones), were identified which displayed GSI sensitive Notch signaling upon transient expression with ZEDN, a rat NotchΔE expression construct [64]. Three clones exhibiting highest signal to noise NICD responsive luciferase activity (SCH-32, SCH-33 and SCH-54) were selected as the hosts for subsequent stable expression of NotchΔE by transfection with pIRES-ZEDN1in the final step of generating a dual-assay cell line. Stable cell lines expressing rat NotchΔE were identified following transfection of the rat NotchΔE construct by selection with media supplemented with the γ-secretase inhibitor ELN-46719 (to suppress Notch/NICD toxicity), G418 (0.5 mg/mL), hygromycin (1 mg/mL) and puromycin (2.5 μg/mL). Antibiotic-resistant colonies (named SNC clones for the dual-assay components: APPsw/NotchΔE/CBF) were isolated and expanded for characterization of Aβ secretion and NICD responsive reporter gene activity (that is, luciferase signal in the presence versus absence of ELN46719). Based on optimal Aβ secretion, and highest signal/background of reporter gene activity, a clone designated as SNC-204B8 was selected as the dual-assay stable line for subsequent profiling of APP selective γ-secretase inhibitors. Activity and selectivity of compounds for inhibition of Aβ production versus Notch signaling was determined by plating the cells in 96-well plates in media lacking ELN46719 overnight. The following day, media was replaced and supplemented one hour later with an equal volume of additional media containing test compounds over a concentration range 0 nM to 40,000 nM (final, in 10X dilution increments). The cells were treated with compounds overnight at 37°C. The following day (Day 3), conditioned media was aspirated from cells for determination of Aβ_1-x _levels by ELISA [65], and cells were lysed for determination of Notch signaling by luciferase reporter gene activity (Promega, Madison, Wisconsin, USA) per manufacturers protocol.

### Analysis of APP metabolites from inhibitor treated cells

APP metabolites were analyzed by Western blot of SDS-PAGE fractionated cell extracts and media derived from HEK293 cells stably expressing APPsw mutation following overnight treatment with different gamma-secretase inhibitors. Equal concentrations of protein (BioRad, Hercules, California, USA) from cell extracts were loaded for Western blot analysis. As reference GSI's we used LY411575, and the Merck active site directed isostere L-685458 for comparison with ELN-318463. Compounds were tested at two doses, approximately 1 X ED_50 _and approximately 10 X ED_50 _values in the SNC assay. Different anti-APP antibodies were used to test the compounds for effects on Aβ, total secreted APP, β-sAPP, full length-APP and APP CTFs (C-terminal fragments of APP resulting from α- and β-secretase cleavage) as noted below. Secreted Aβ in the conditioned media was quantified by ELISA using 2G3/3D6, which detects Aβ (1 to 40). The full length APP and CTF Western blots were probed with anti-APP-c-terminal specific antibody (Sigma). Western blots of the conditioned medium samples (normalized for differences in cell density by protein concentration of the cell extracts) were probed with antibody 8E5 [66] which recognizes total secreted APP, and 192sw [67,68] which recognizes the carboxy-terminus of secreted APP arising from BACE cleavage of the APPsw overexpressed in these HEK293 cells.

### Binding site studies

A displacement assay employing affinity capture of gamma-secretase complex with a biotinylated analog of active site isostere L685,458 [69] was developed to characterize binding site of the APP selective sulfonamides. PS1 and Nicastrin (Nct) Western blots were employed as readout for association of the gamma-secretase complex with the biotinylated probe. Displacement of the active site isostere by test compounds resulted in lowered intensity of PS1 and Nct bands on the immunoblots. The biotinylated active-site isostere inhibited APP cleavage in our gammaAPP assay with an IC50 = 11 nM. The results with L685,458 and LY-411575 were repeated three times, while the result observed with ELN318463 was replicated with a close structural analog of ELN318463 (not shown). The result shown with ELN475516 is from one of two independent replicates. Briefly, in the displacement assay a WGA affinity purified solubilized membrane preparation containing enriched gamma-secretase enzyme (from CHO S1 cells [70] prepared as described above), is diluted in incubation buffer under gammaAPP reaction conditions, and pre-cleared (30 minutes at room temperature) with streptavidin-sepharose resin (Amersham/GE Healthcare, Piscataway, New Jersey, USA) in order to remove endogenous biotinylated proteins and reduce nonspecific background signal. Biotinylated analog at 50 nM is added to pre-cleared extract with or without competing test compounds at different concentrations, and incubated at 37°C for two hours. Streptavidin-sepharose is then added, and the mixtures are incubated at RT for 30 minutes. Following capture, the beads are centrifuged briefly (two minutes at 10,000 g) and washed twice with the incubation buffer, followed by Western blot analysis (Immobilon membranes, Millipore, Billerica, Massachusetts, USA) for PS1 NTF (antibody 1563, Chemicon, Millipore Bioscience Research Reagents, Temecula, California, USA) and Nicastrin (antibody N1660, Sigma, St Louis, Missouri, USA) specifically associated with the streptavidin resin through the biotinylated compound. The samples are run on 26-well 10% to 20% acrylamide Tris-HCl gels (Criterion, BioRad), alongside serially diluted affinity captured enzyme samples from the same experiment (minus competing test compound) to provide a standard curve of on each gel for quantitation purposes. The PS1 and Nicastrin signals were detected by horse-radish peroxidase labeled secondary antibody followed by ECL chemiluminesence (Amersham) and the images were quantified by densitometry. The relative amount of PS or Nicastin remaining associated with affinity ligand in the unknown samples is calculated from the standard curve, and displacement of the biotinylated compound is reported as percentage of the protein signal in DMSO vehicle control samples. Binding assays in the presence of substrate were performed exactly as described above with the inclusion of 20 μg/ml (approximately 1 Km) of MBP-APPc125 recombinant fusion protein [61]. Preliminary experiments (not shown) indicated that under these conditions, we are able to capture >90% of enzyme complex in the mix, and that >90% of captured complex is competed by co-incubation of the binding mix with 10 μM L-685,458.

### Acute reduction of brain Aβ in FVB and PDAPP mice

All experiments were approved by the Institutional Animal Care and Use Committee (IACUC) of Elan Pharmaceuticals and conducted in accordance with its guidelines. Female, two- to three-month old, FVB/N mice were purchased from Taconic (Oxnard California, USA). Mice were housed four to five to a cage on a 12-hour light/dark schedule. Water and food were available *ad libitum*. FVB mice were treated orally with a single dose (*n *= 7/dose) of compound as indicated in the associated Figures. The test articles were formulated at various concentrations in a 10% Solutol^®^/water vehicle (BASF Corp, Florham Park, New Jersey, USA) and dosed at 5 mL/kg via oral gavage. Control animals were dosed with vehicle. Animals were sacrificed at three hours post dose, unless indicated otherwise. Cortical brain samples were collected, frozen on dry ice and stored at -80°C until homogenization for determination of Aβ and compound levels. Thymus was collected, frozen in liquid nitrogen, stored at -80°C for analysis of Hes1 levels. Blood samples were collected by cardiac puncture, processed to plasma, frozen on dry ice and stored at -80°C until determination of compound levels by LC-MS-MS. Statistical analysis of data was performed using one-way ANOVA followed by Dunnett's.

### Repeat dose mouse seven-day toxicity study

FVB mice, female, two to three months old, were treated orally with ELN475516 (15, 30, 100, 300, 600, and 1000 mg/kg/dose) or LY411575 (5 mg/kg/dose) twice per day (b.i.d.) for seven days (*N = *5 per treatment group). The test articles were formulated at various concentrations in a 10% Solutol^®^/water vehicle and dosed at 5 mL/kg via oral gavage. Control animals were dosed with vehicle. All animals were sacrificed at three hours post last dose. Cortical brain samples were collected, frozen on dry ice and stored at -80°C until homogenization for determination of Aβ and compound levels. Blood was collected, (via cardiac puncture), processed for CBC, or processed to plasma and frozen on dry ice and stored at -80°C until determination of compound levels. Duodenum and ileum, as well as other tissues, were collected from each treatment group and fixed in 10% formalin for histopathology evaluation. Body weight was reported as percent change from the base line and compared to vehicle control group in the one-way ANOVA Dunnett's test for statistical analysis. Relative organ weight was calculated by the absolute organ weight/body weight. Statistical analysis was done by Prism software/one-way ANOVA (and nonparametric) Dunnett's test for all toxicity endpoints. Most endpoints examined were significant at *P *< 0.001 except on one parameter which was *P *< 0.01 and is noted in the appropriate figure legend. Duodenum and ileum tissues were sectioned and double stained with H&E (hematoxylin and eosin) plus PAS (periodic acid-Schiff stain) for histopathologic evaluation.

### Endogenous mouse brain Aβ ELISA

Cortical brain samples were homogenized in 5 M guanidine and Aβ_x-40 _was estimated in a sandwich ELISA utilizing the monoclonal antibodies 266 (made against 16 to 28 amino acid sequence of Aβ) as the capture, and 2G3 (terminal specific to Aβ40) as the reporter. This assay estimates the concentration of Aβ peptides ending at amino acid 40, without specificity for amino termini upstream of residue 16. Statistical significance was determined by ANOVA.

### Quantitation of Notch signaling RNA endpoints Hes1/Math1

Tissues were homogenized in lysis buffer and processed for total RNA extraction using the QIAGEN Rneasy 96-well plate technology with post-column Dnase treatment. The concentration of RNA per sample was measured by a RiboGreen RNA quantitation assay (Invitrogen). Math1 (Atonal homolog) or Hes1 mRNA levels were quantified by a TaqMan RT-PCR assay and results were normalized by RNA sample concentration. The sequences of the primers and probes employed in the Taqman assay were as follows: Hes1 forward primer: CGGCTTCAGCGAGTGCAT; Hes1 reverse primer: CGGTGTTAACGCCCTCACA; Hes1 probe: AACGAGGTGACCCGCTTCCTGTCC. Math1 Forward primer: AGCTGGACGCTTTGCACTTT; Math1 reverse primer: TCTGTGCCATCATCGCTGTT; Math1 Probe: 5'FAM-CAGCTTTCGAGGACCGGGCCC-TAMRA3'. All Taqman reagents were acquired from Applied Biosystems, Inc., Foster City, CA, USA.

## Results

**Figure 1:**
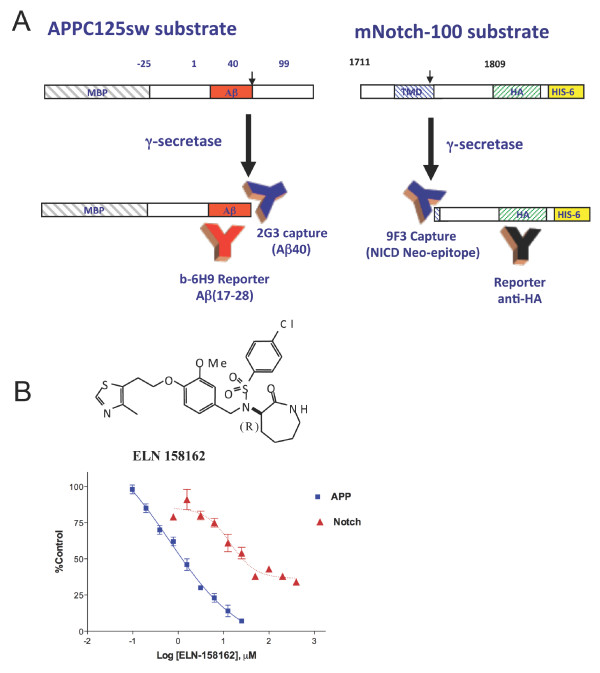
**Schematic illustration of in-vitro assays, and dose response curves of an APP selective gamma-secretase inhibitor**. **(a) **Recombinant *E.coli *expressed fusion protein substrates APP MBP-C125Sw (left side, numbering relative to Aβ peptide residues, red box) or mNotch100 substrate (right hand side, numbering relative to initiator methionine residue) were incubated in separate reactions with partially purified solubilized gamma-secretase enzyme from IMR-32 cells as detailed in methods. The reactions products were analyzed by ELISA using 2G3/6H9 ELISA for Aβ_X-40 _for GammaAPP assay product, or the 9F3/anti-HA ELISA for NICD, the GammaNotch assay product. MBP, N-terminal fusion with maltose binding protein (grey hatched box); TMD, transmembrane domain (blue hatched box); HA, hemagglutnin antigen epitope tag (green hatched box); HIS-6, hexahistidine residue epitope tag (yellow box). **(b) **Dose response curve demonstrating APP-selective inhibition from a representative screening hit ELN158762. The IC_50 _values for inhibition of APP and Notch substrate cleavage, and selectivity ratio in the biochemical assays with this compound are reported in Table 1.

To enable discovery of novel APP selective gamma-secretase inhibitors, we developed biochemical assays for substrate cleavage followed by ELISA employing neo-epitope specific monoclonal antibodies for detection of products. A detergent solubilized, lectin affinity column enriched, enzyme preparation derived from IMR-32 neuroblastoma cells was incubated with either recombinant APP-C125, or a Notch fusion protein substrate. These substrates replicate natural processing of a site equivalent to the carboxy-terminus of Aβ40, or the S3/ε-site of Notch, respectively, with Michaelis-Menten kinetics (APP substrate K_m_= 358 nM, Notch substrate K_m _= 1.52 μM, Figure S1 in Additional file 1). The products of this reaction were detected and quantified by a sandwich ELISA employing neo-epitope specific capture monoclonal antibodies 2G3 (Aβ40 specific), or 9F3 (specific for NICD N-terminus, the C-terminal cleavage product derived from NotchΔE in cells [71-73], Figure S2 in Additional file 2), and appropriate detection antibodies as illustrated in Figure 1a. Henceforth, we refer to this assay measuring gamma-secretase cleavage of APP fusion protein substrate at Aβ40 site as the gamma:APP assay, and cleavage of NotchΔEΔC substrate at Notch S3/ε-site as gamma:Notch assay.

A high-throughput screen of a chemical library employing the gamma:APP assay was conducted to discover novel inhibitors of gamma-secretase. Hits from the primary screen (defined as >25% inhibition at 10 μM) were characterized in secondary assays for dose response, non-specific inhibition (for example, of non-gamma:APP enzymatic reactions, assay detection reagents), and selectivity for inhibition of APP cleavage versus Notch cleavage by gamma-secretase. A number of hits comprising a novel series of p-chlorobenzene-caprolactam sulfonamide compounds demonstrated concentration dependent and APP > Notch selective inhibition of gamma-secretase, as exemplified by ELN158162 , which displayed an IC_50 _in gammaAPP assay of 0.65 μM, compared with an IC_50 _of approximately 25 μM in the gamma:Notch assay, resulting in a selectivity ratio of approximately 40 (Figure 1b).

To test for APP-selective inhibition of gamma-secretase by the screening hits in a cellular context, we developed a stable CHO cell line co-expressing APPSw, rat NotchΔE [64], and an NICD responsive CBF-Luciferase reporter gene [63] (SNC cells), to assay endogenous gamma-secretase enzyme. Inhibition of secreted Aβ was quantified by an ELISA specific for Aβ_1-x _using conditioned media harvested from cells, and Notch signaling was detected using a luciferase reporter assay in cellular lysates from cells treated overnight with a concentration range of inhibitor compounds. The profile of reference non-selective gamma-secretase inhibitors compared with the HTS hit and selective lead compounds in the SNC dual cell assay corroborated the biochemical enzymatic assay data, demonstrating APP selective inhibition of cellular gamma-secretase by the screening hit and select lead compounds (Table 1).

**Table 1:** In-vitro Properties of Gamma-Secretase Inhibitors

Compound	gamma:APP IC50 (nM)	gamma:Notch IC50 (nM)	enzyme selectivity (APP/Notch)	SNC Aβ EC50 (nM)	SNC Notch Signaling EC50 (nM)	Cellular Selectivity (APP/Notch)
L-685,458	0.63 (1)	nd	NA	13.2 ± 4.5 (4)	184 ± 145 (3)	14
LY411575	0.035 (1)	0.082 ± 0.008 (3)	2.4	0.116 ± 0.011 (3)	1.8 ± 1.35 (3)	16
ELN158162	650	14,000	21.5	nd	nd	NA
ELN318463	37.2 ± 18 (51)	1,889 ± 722 (3)	51	23.4 ± 8.6 (10)	2,818 ± 670 (10)	120
ELN475516	2.06 ± 0.35 (16)	29.81 ± 2.59 (4)	14.5	8.73 ± 3.96 (298)	719 ± 224.6 (298)	82

Potency For Inhibition Of App And Notch Processing by select gamma-secretase inhibitors in enzyme, as well as cellular assays described in text. The value in parentheses next to each IC50 indicates the number of independent test occasions used to derive the IC50 value. Note: Cellular selectivity of L-685,458 was determined prior to development of the SNC assay using different cellular assays for Aβ production and Notch signaling.

Abbreviations: APP, amyloid precursor protein; SNC, a stable CHO cell line co-expressing APPSw, rat NotchΔE, and an NICD responsive CBF-Luciferase reporter gene used to simultaneously assay inhibition of Aβ production, and Notch signaling; Aβ, amyloid beta-peptide.

**Figure 2:**
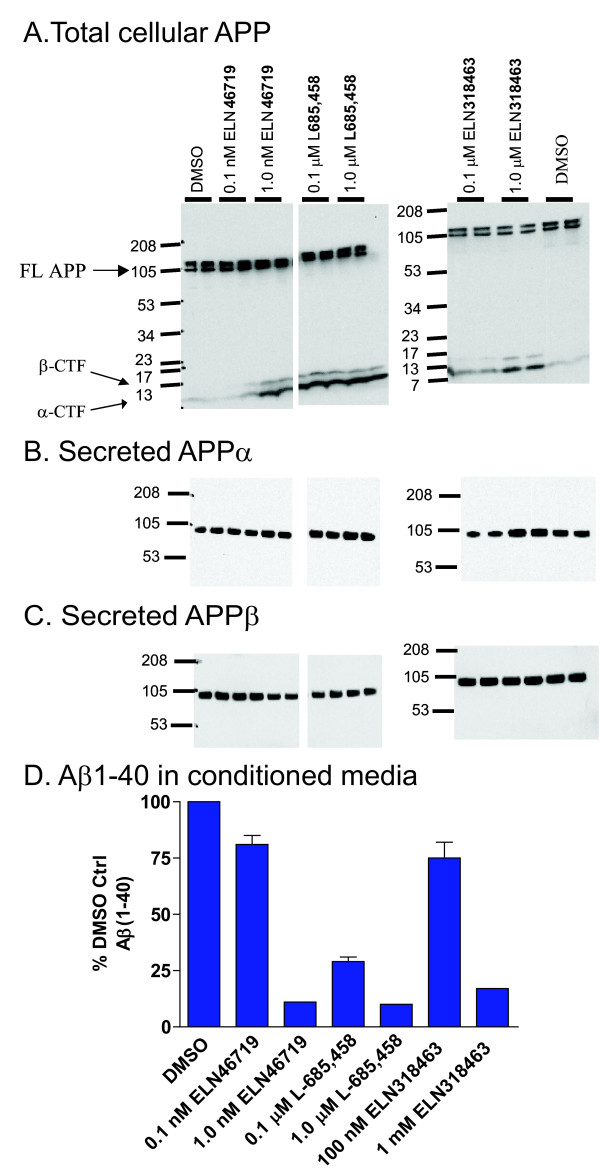
**Novel sulfonamides exhibit properties in common with classic gamma-secretase inhibitors**. Western blots of cell extracts **(a) **and media **(b, c) **after treatment of cultures in duplicate of with gamma-secretase inhibitors indicated above the lanes. Compounds were tested at two doses, approximately 1 X ED_50 _and approximately 10 X ED_50 _based on cellular assay results. (a) Full length and c-terminal fragments of APP total cellular lysates recognized by an antibody against the C-terminus of APP (Sigma A8717). The reference gamma-secretase inhibitors L-685,458 and ELN46719 (analog of LY411575, see methods), as well as the novel sulfonamides tested stabilize both α-CTF and β-CTF in a dose-dependent manner, and none of the compounds significantly affect steady-state levels of full-length APP. (b, c) Western blots of the conditioned media samples from this same experiment probed with antibody 8E5 against total secreted APP (b), and 192Sw against the β-secreted APP formed by BACE cleavage of the Swedish mutant APP over-expressed in these HEK293 cells (c). No significant effect on either total sAPPα or sAPPβ was seen by any of the gamma-secretase inhibitors. (d) Quantification of Aβ_1-40 _in the conditioned media. The results suggest that the sulfonamide only affects gamma-secretase cleavage of APP, and does not appear to have nonspecific effects on overall APP metabolism or cell viability in HEK293 cells.

**Figure 3:**
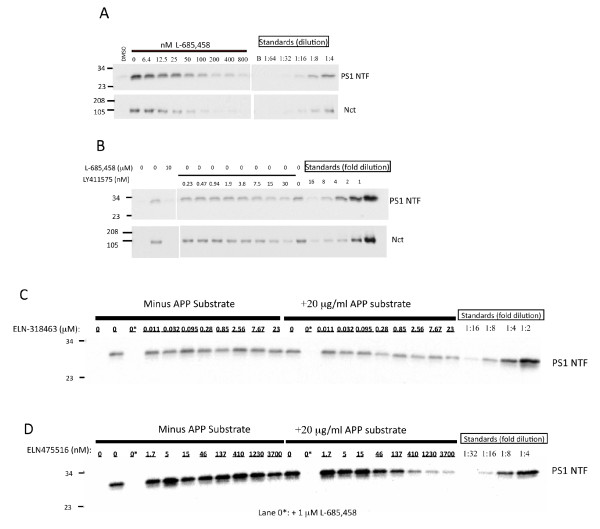
**Binding site analysis by competitive displacement of biotinylated active site isostere**. A competitive binding assay employing a biotin conjugated active site isostere to capture gamma secretase from a partially purified cell extract, plus or minus co-incubation with increasing concentrations of reference or test gamma secretase inhibitors, was developed to characterize inhibitor binding site. The eluted enzyme captured on neutravidin beads was detected on western blots with antibodies recognizing either PS-1 amino-terminal fragment (PS1-NTF), or Nicastrin (Nct). The binding assay was carried out using 50 nM affinity probe together with different concentrations of competing compounds. Serial dilutions of affinity captured enzyme were included on the gels to provide a standard curve for densitometric quantitation of test samples following autoradiography of the western blots. **(a) **The biotinylated isostere is displaced by its non-biotinylated analog L-685,458 in a concentration dependent manner. In subsequent experiments, a 200-fold excess of L-685,458 was employed as a positive control. **(b) **LY411575 was tested for its ability to displace active site isostere at concentrations ranging up to 1000-fold its IC_50 _in the Gamma APP enzyme assay. There is no significant displacement of the active site directed compound by at concentrations below 200-fold its IC_50 _in the enzyme assay. At higher concentrations, a modest dose-dependent effect of LY411575 to displace the active-site-directed compound is observed on both Western blots. Previous observations revealed substrate concentration affects the potency of sulfonamides in cell and enzyme assays (unpublished). Hence, the ability of sulfonamides to displace the active site directed probe was tested in the absence **(c and d**, left side**) **or presence (c and d, right side) of 1 Km substrate (MBP-C125sw). Substrate was added to enzyme concurrently with test compound and affinity ligand. In the presence of added APP, the sulfonamides displace the active site probe in a dose-dependent manner. (c) No displacement of active site probe is observed by ELN-318463 in the absence of substrate. In the presence of substrate, ELN318463 displaces approximately 50% of the active site probe at a concentration of approximately 2,000-fold its IC_50 _in the Gamma APP assay. (d) ELN-475516 does not displace the active site probe at concentrations ranging up to 2,000X its IC_50 _in the gammaAPP assay. However, in the presence of substrate, ELN475516 displaces 50% of the active site probe at a concentration equivalent to approximately 67-fold its Gamma APP IC_50_. The results shown in **(c) **and **(d) **suggest displacement of active site isostere from gamma-secretase by benzene caprolactam and fused pyrazolo-bicyclic sulfonamide is influenced by the presence of substrate.

Biochemical characterization of the inhibitors revealed they behave as classical GSIs in that they elevate C99 with no effects on sAPPα or sAPPβ (Figure 2), and are equipotent inhibitors of Aβ40 and Aβ42 (Figure S3 in Additional file 3). The compounds lowered secreted Aβ in conditioned media at the concentrations tested for effects on APP metabolites (Figure 2d). Binding site studies employing biotin labeled active site isostere as affinity ligand for enriched gamma-secretase enzyme (derived from CHO-S1 cells [70], flow-chart of assay shown in Figure S4 in Additional file 4) confirmed competitive displacement of affinity ligand by its non-biotinylated analog L-685,458 (Figure 3a), consistent with published results [69], as well as by very high concentrations of LY411575 (Figure 3b). Specifically, we observed a 50% displacement of a biotinylated active site isostere probe by L-685,458 at concentrations approximately three-fold above its IC_50 _in the gammaAPP assay, whereas LY-411575 displaced 50% of biotinylated active site isostere probe at concentrations 500X to 1,000X above its IC_50 _value in the gammaAPP assay. Sulfonamides did not displace biotinylated active site isostere in competitive binding assays in the absence of substrate (Figure 3c, d, left half). However, in the presence of 1 K_m _(20 μg/ml) MBP-C125 substrate, ELN318463 and ELN475516 were able to displace the active site isostere (Figure 3c, d, right half) at an ED_50 _of 23 μM and 0.14 μM, respectively (data not shown). These values represent an approximately 2,000X and 67X multiple over the IC_50 _values of the two compounds in the gammaAPP assay.

**Figure 4:**
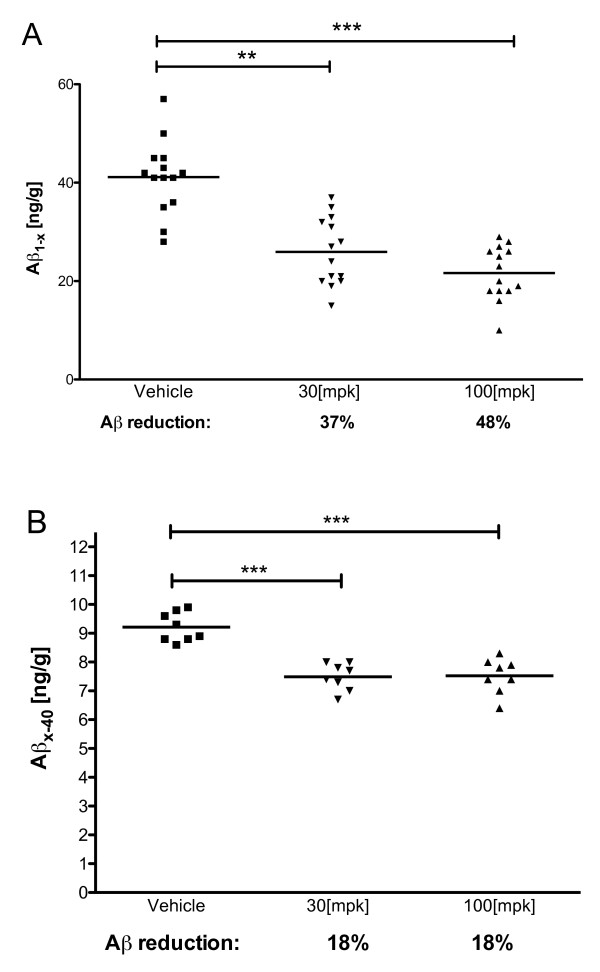
**Acute reduction of brain Aβ by ELN318463 in PDAPP (a) and FVB mice (b)**. The mice were dosed orally with vehicle, or ELN318463 at 30 mg/kg or 100 mg/kg and sacrificed 3 h post treatment. Brain Aβ_1-x _in PDAPP (a), or Aβ_x-40 _in FVB (b) was quantified by ELISA following extraction in guanidine buffer as described in methods. ** denotes *P *< 0.01, *** denotes *P *< 0.001 by Mann-Whitney in A, and two-tailed Students *t*-test in (b).

*In vivo *testing of a benzene caprolactam sulfonamide, ELN318463 [59], possessing favorable oral bioavailabilty (F = 30% in rat), revealed dose-dependent acute reduction of brain Aβ_1-x _species in PDAPP mice (Figure 4a). In contrast, acute reduction of Aβ_x-40 _species in non-transgene FVB mice (Figure 4b) was equivalent at both doses tested. Reduction of Aβ_x-40 _in PDAPP also was not dose responsive (20% and 25% reduction at 30 mg/kg and 100 mg/kg, respectively, data not shown). The lack of a dose response in Aβ_x-40 _species in PDAPP & FVB mice compared with Aβ_1-x _species in PDAPP mice can not be explained by differences in terminal compound levels in the target organ at the two doses tested, nor by assay differences. Brain levels of ELN318463 at 30 mg/kg were 0.754 μM in FVB brains and 0.69 μM in PDAPP brains, and at 100 mg/kg dose the levels were 2.7 μM in FVB brains and 1.87 μM in PDAPP brains. Discordance between Aβ_1-40 _and Aβ_x-40 _in BACE KO or wild-type BACE inhibitor treated mice has been reported [74]. However, our observations cannot be explained by differences in assays employed for measuring Aβ_1-x _vs. Aβ_x-40_, as observed by Nishitomi *et al. *[74], because both assays used in this study are insensitive to the Aβ P3 fragment (product of alpha- and gamma-secretase cleavages) by virtue of the capture antibody employed (see Materials and methods).

**Figure 5:**
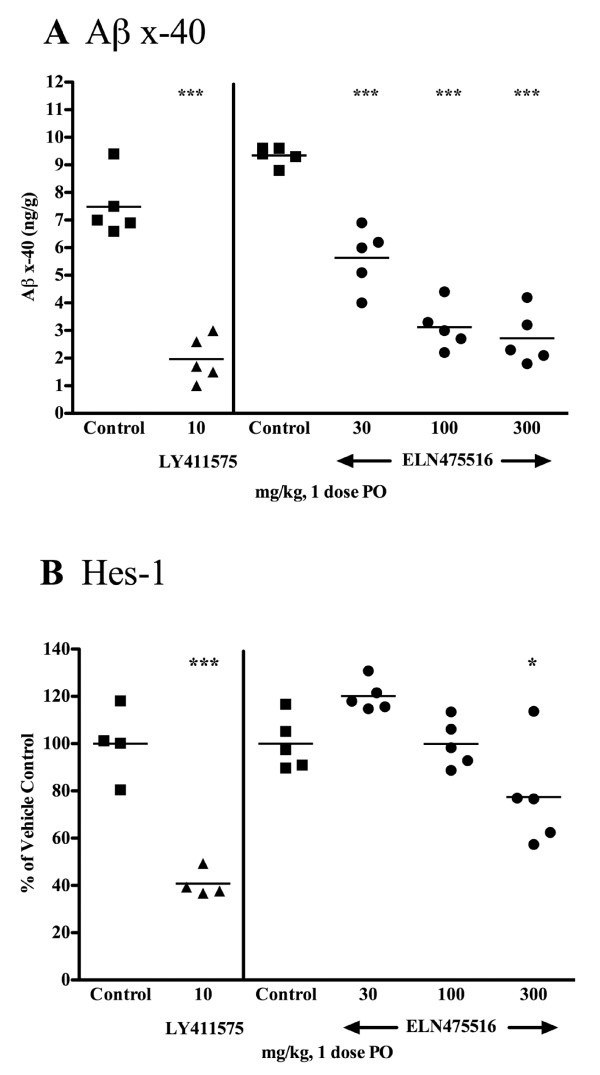
**Reduction of brain Aβ and thymic Hes-1 in wild-type FVB mice after treatment with gamma-secretase inhibitor**. Female FVB mice were treated orally with a single dose of ELN475516 (30, 100 or 300 mg/kg), LY411575 (10 mg/kg) or vehicle and sacrificed at three hours post dose. **(a) **Cortical Aβ_x-40 _levels, estimated by ELISA from guanidine extracted brain homogenates. **(b) **Thymic Hes-1 mRNA levels, estimated by TaqMan RT-PCR. Statistical significance between treatment groups and vehicle was determined by ANOVA and Dunnett's test. *** *P *< 0.001, * *P *< 0.05

**Figure 6:**
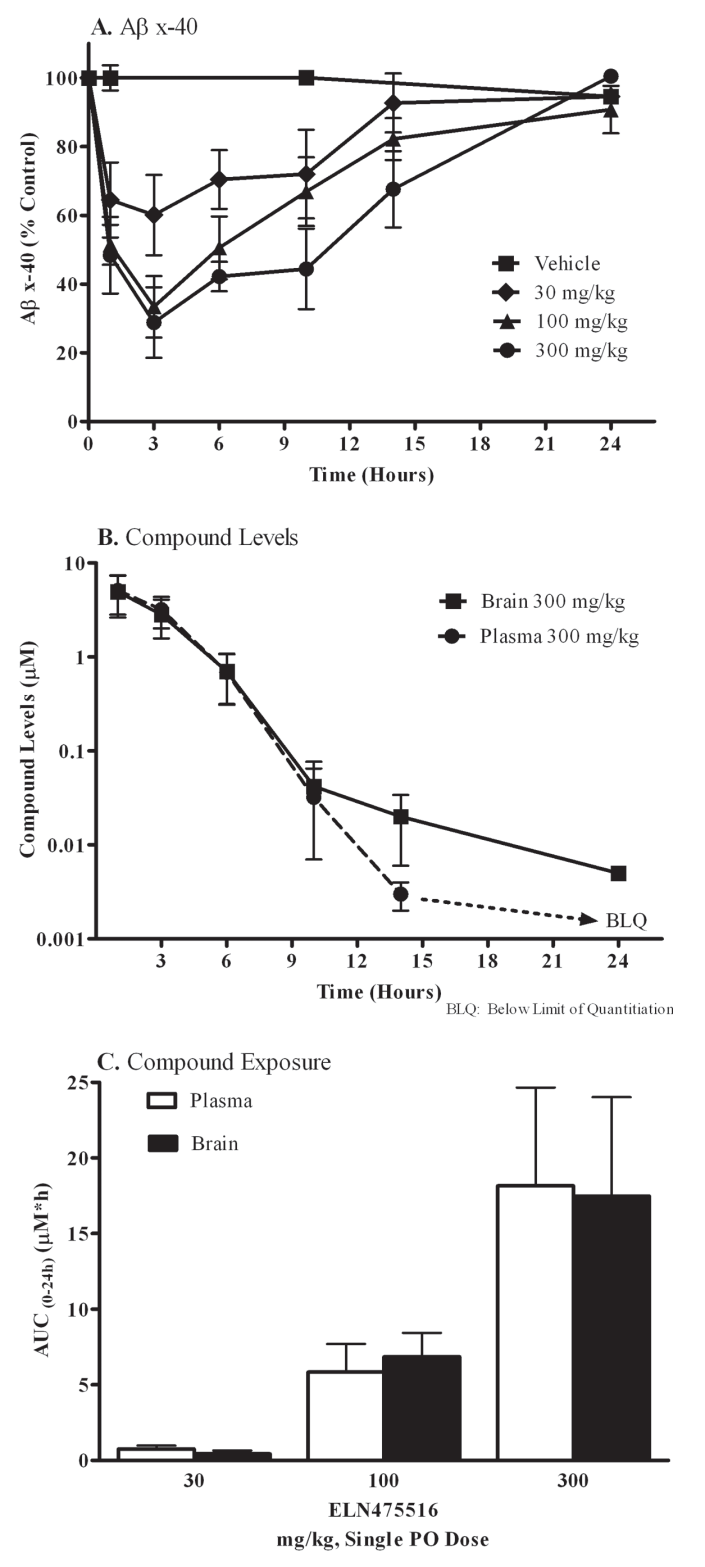
**Time course of brain Aβ reduction in wildtype FVB mice after treatment with a single dose of ELN475516**. Female FVB mice were treated orally with a single dose of ELN475516 (30, 100 or 300 mg/kg, *n *= 5/dose group) or vehicle and sacrificed at 1, 3, 6, 10, 14 and 24 hours post dose. **(a) **Cortical Aβ_x-40 _levels, estimated by ELISA from guanidine extracted brain homogenates. **(b) **ELN475516 levels in plasma and brain estimated by LC/MS/MS. **(c) **ELN475516 plasma and brain exposure over 24 hours (AUC_0-24_).

**Figure 7:**
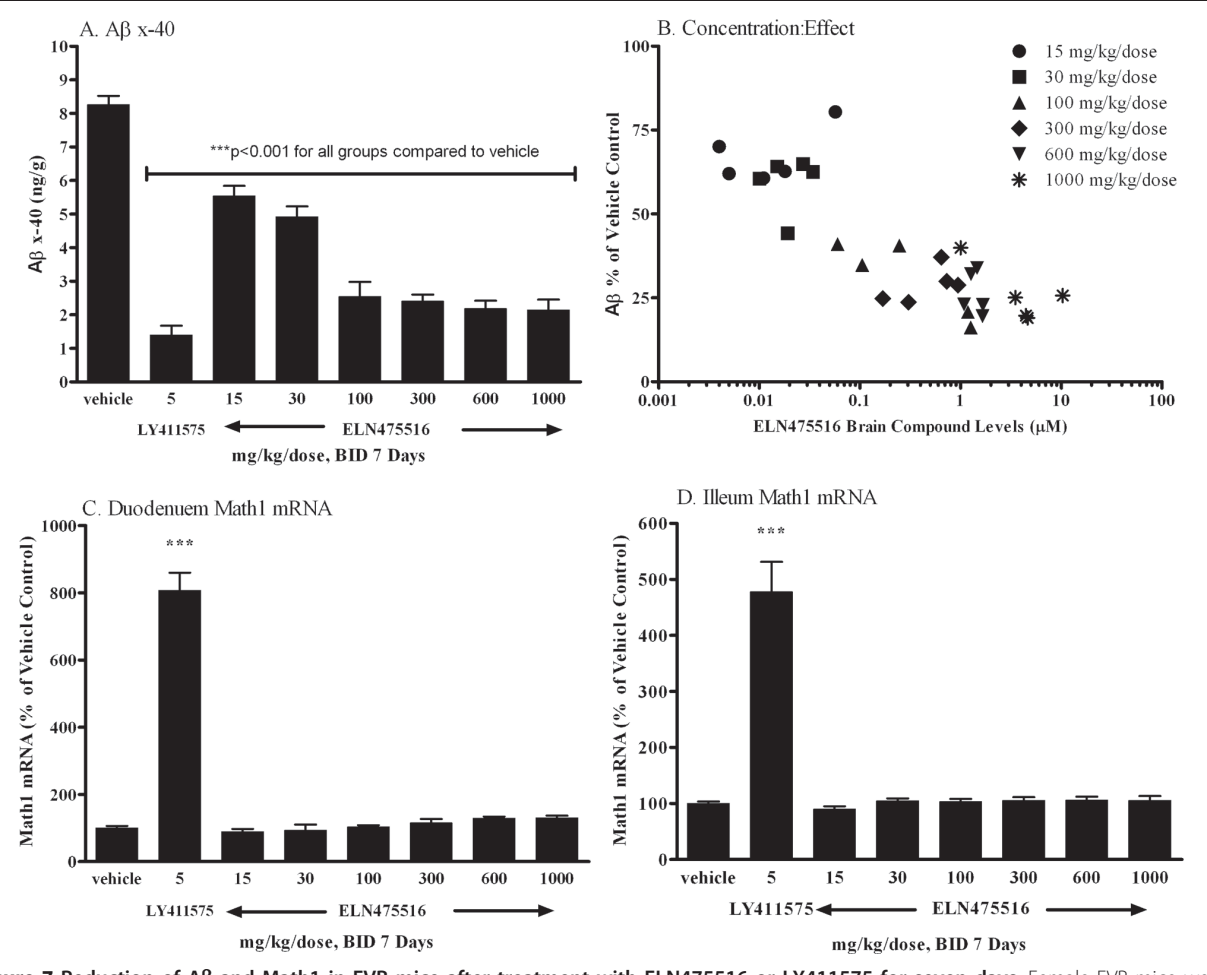
**Reduction of Aβ and Math1 in FVB mice after treatment with ELN475516 or LY411575 for seven days**. Female FVB mice were treated orally twice daily with ELN475516, LY411575 or vehicle and sacrificed at three hours post last dose. **(a) **Cortical Aβ_x-40 _levels, estimated by ELISA from guanidine brain homogenates. Statistically significant (*P *<.0001) Aβ reduction (33%) was achieved at the lowest dose tested (15 mg/kg/dose). **(b) **Relationship of ELN475516 levels in plasma (estimated by LC/MS/MS) vs. brain Aβ_x-40 _levels (estimated by ELISA from guanidine brain homogenates). Duodeneum **(c) **and Ileum **(d) **Math1 mRNA levels, estimated by TaqMan RT-PCR. Treatment with 5 mg/kg/dose LY411575 BID for seven days resulted in a robust elevation of Math1 gene expression in the duodeneum and ileum (*P *< 0.001). No effects on Math1 in the duodeneum or ileum were observed after treatment of ELN475516 BID for seven days up to the highest dose tested, 1,000 mg/kg/dose (2,000 mg/kg/day). Statistical significance between treatment groups and vehicle was determined by ANOVA and Dunnett's test. ***, *P *< 0.001

Lead optimization toward discovery of more potent analogs revealed critical requirement for proton donor in caprolactam ring of benzene-sulfonamides, corroborated by pyrazole substituted piperidines as scaffold replacements [60] (Figure S5 in Additional file 5). The prototype pyrazolylazabicyclo(3.3.1)nonane ELN475516 supported this hypothesis by demonstrating improved *in vitro *potency, while retaining APP selectivity of the caprolactam sulfonamide parent lead series (Table 1). Furthermore, the novel fused bi-cyclic gamma-secretase inhibitor demonstrated dose dependent *in vivo *activity for acute reduction of cortical Aβ_1-x _in the PDAPP transgene mouse model (31% and 45% reduction of Aβ_1-x _at three hours following a single oral dose of 10 mg/kg, and 30 mg/kg, respectively, data not shown) as well as cortical Aβ_x-40 _in wild type FVB mice (Figures 5a, 6a, and 7a).

In acute single dose studies, ELN475516 lowered brain Aβ at three hours post treatment in a dose dependent manner, effecting a 40% reduction at the lowest dose tested (30 mg/kg), and up to a 71% reduction at 300 mg/kg (Figure 5a). A statistically significant reduction of thymic Hes-1 mRNA (an acute end-point of Notch signaling inhibition) was detected at the highest dose tested (300 mg/kg. Figure 5b). Terminal plasma compound levels at the highest dose tested were 12.3 μM, or a more than six-fold multiple over the cellular IC_50 _for inhibition of Notch signaling. By comparison, acute treatment with10 mg/kg of the non-selective GSI LY411575 concomitantly lowered brain Aβ (74%) and thymic Hes-1 mRNA (60%) (Figure 5a, b, respectively). The acute treatment study shown in Figure 5 provides a benchmark for reduction of brain Aβ in FVB mice treated with 30 mg/kg ELN475516 at three hours post dose.

To characterize the duration of efficacy in relation to compound exposure at different doses for ELN475516, we conducted a time course study in mice treated with 30, 100, and 300 mg/kg, and terminated at varying time points following single dose. The results, shown in Figure 6, indicate that Cmax was achieved at one hour post dose (Figure 6b), maximal reduction of brain Aβ was observed at three hours post dose (Figure 6a), and significant reduction was maintained at 10 hours post dose at all doses tested (28%, 33%, and 56%, at 30, 100, and 300 mg/kg, respectively). At 14 hours post dose, a significant 14% and 29% reduction was observed in mice treated with 100 and 300 mg/kg, with Aβ levels returning to baseline by 24 hours post dose in all treatment groups. The results shown in Figure 6a suggest that brain Aβ was lowered by >25% for 24 hours per day in mice dosed >300 mg/kg. Dose dependent increases in brain and plasma exposure were seen in mice at the three doses tested in this single dose study, where total exposure in the 300 mg/kg group was 24X the 30 mg/kg group, and 3X the 100 mg/kg group (Figure 6c).

**Figure 8:**
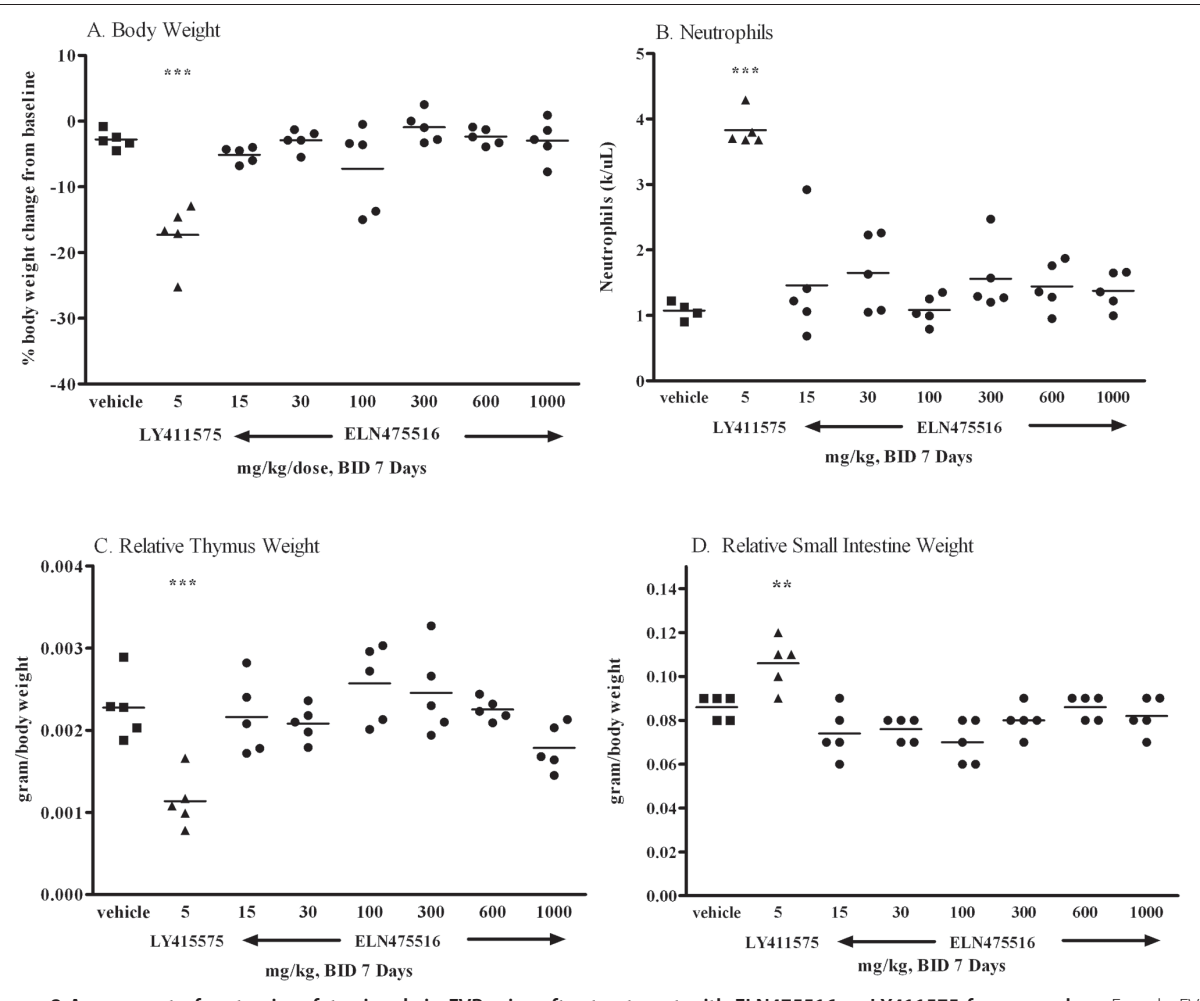
**Assessment of systemic safety signals in FVB mice after treatment with ELN475516 or LY411575 for seven days**. Female FVB mice were treated orally twice daily with ELN475516, LY411575 or vehicle and sacrificed at three hours post last dose. Mice treated with 5 mg/kg LY411575 BID for seven days showed consistent signs of toxicity while there were no observed signs of toxicity in any group treated with ELN475516, even up to the highest dose of 1000 mg/kg (2000 mg/kg/day) for seven days. **(a) **Body weight **(b) **Neutrophils **(c) **Relative thymus weight and **(d) **Relative small intestine weight. Statistical significance between treatment groups and vehicle was determined by ANOVA and Dunnett's test. *** *P *<0.001, ** *P *< 0.01.

**Figure 9:**
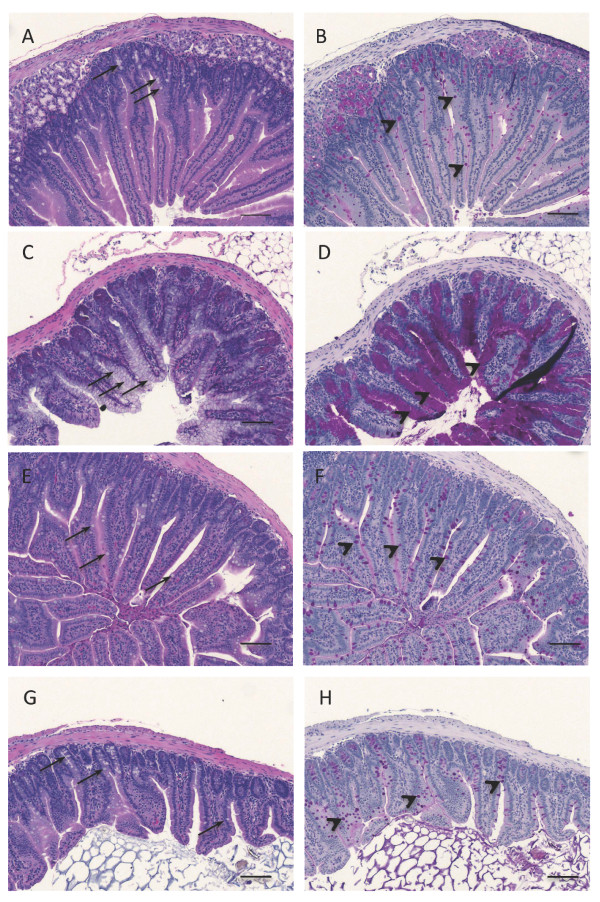
**Photomicrographs of ileum sections (10X magnification) from mice treated seven days x b.i.d. with gamma-secretase inhibitors noted below**. Selected sections represent the range of goblet cell hyperplasia observed in this study. In H&E stained sections **(a, c, e and g)**, goblet cells appear as cells with clear or foamy cytoplasm (arrows) while in PAS stained sections **(b, d, f and h) **they appear as dark magenta stained cells (arrow heads). A normal population of goblet cells was observed in all vehicle control mice (a, b). All positive control LY411575 treated mice had a moderate increase in goblet cells (c, d). There was no apparent dose-related increase in goblet cells across ELN475516 treated groups (see Table 2); among all mice in these groups, 21 had no increase in goblet cells, 8 had a questionable increase (illustrated by a single animal from 100 mg/kg dose group g, h). A single mouse from the 600 mg/kg dose group had a mild increase in goblet cells, which is shown for comparison purposes in panels (e and f). Scale bar = 100 μm.

To provide a critical test of improved *in vitro *selectivity in an *in vivo *context, we developed a seven-day mouse safety model utilizing a b.i.d dosing regimen in wild-type FVB mice. Mice treated with a non-selective GSI (LY411575) at 5 mg/kg b.i.d. demonstrated 83% reduction of brain Aβ (Figure 7a). However, the reduction of brain Aβ at this dose was accompanied by concomitant effects on endpoints related to Notch signaling in peripheral organs. These effects included a decrease in body and thymus weight, increase in circulating neutrophils, goblet cell hyperplasia, plus elevated Math1 mRNA endpoints in duodenum and ileum indicative of Notch signaling inhibition, as shown in Figure 7c, d, Figure 8 and Figure 9c, d. Hence, the mouse seven-day b.i.d. treatment model accurately reflects systemic as well as target organ toxicities described for this non-selective GSI, consistent with reports in other models [28,29,75].

Based on the observed acute safety and pharmacokinetics (Figures 5 and 6), FVB mice were treated twice daily with a range of oral doses of ELN475516 for seven days as described in methods. The pharmacological and safety end-points following this seven-day treatment are shown in Figures 7, 8 and 9. Treatment of mice with ELN475516 at the lowest dose tested (15 mg/kg/dose, b.i.d.) resulted in a statistically significant 33% reduction of brain Aβ (at three hours post last dose), in close agreement with the acute treatment study. The pharmacological effect of ELN475516 on brain Aβ levels in FVB mice was dose dependent, with maximal effect (74% reduction) observed at >100 mg/kg, and exhibited a clear pharmacokinetic/pharmacodynamic relationship in which brain compound levels correlated inversely with brain Aβ levels (Figure 7b). In contrast with a statistically significant effect of the non-selective GSI LY411575 on molecular Notch signaling endpoints (Math-1 mRNA levels in duodenum and ileum, Figure 7c, d), systemic Notch target organ related endpoints (thymus and small intestine weights relative to body weight, Figure 8c, d), as well as systemic endpoints of general toxicity (body weight, circulating neutrophils, Figure 8a, b), ELN475516 had no significant effect relative to vehicle treated controls on these endpoints at all doses tested.

We assessed cell homeostasis in the intestinal tract as a more sensitive indicator of *in vivo *inhibition of Notch signaling by gamma-secretase inhibitors. Goblet cell hyperplasia was readily apparent in mice treated with 5 mg/kg of non-selective GSI LY411575 in both the duodenum and ileum of the intestinal tract (Table 2, Figure 9c and 9d), consistent with published observations [28,29,41,75]. Goblet cell hyperplasia was not observed at any dose of ELN475516 in the duodenum. In the ileum, although scattered minimal to mild goblet cell hyperplasia was observed in a subset of animals from some dose groups (Figure 9e-g), the changes in goblet cell numbers observed were not dose-related. The lack of effect on gastrointestinal tract cell homeostasis in mice treated with up to 1,000 mg/kg/dose ELN475516 b.i.d. is consistent with lack of effect on molecular and systemic readouts of Notch signaling shown in Figures 7 and 8. Terminal plasma levels of compound in the lowest and highest treated dose were 0.187 μM, and 12.3 μM, respectively. An *in vivo *selectivity of >65 for ELN475516 based on either dose or terminal plasma levels of compound is estimated, where minimal efficacious dose is defined as ≥25% Aβ reduction in the brain at study termination (that is, three hours post last dose), and the minimum toxic dose is defined as the dose producing a statistically significant elevation of Math1 mRNA levels. Hence, the *in-vivo *selectivity of ELN475516 corroborates the improved biochemical as well as cellular selectivity for inhibition of Aβ production from APP by gamma-secretase, compared with inhibition of Notch processing and Notch signaling. Limiting N-glucuronidation precluded longer-term studies with ELN475516 in mice (or other models). However, ELND006, a lead optimized analog derived from ELN475516, was well tolerated for three and six months in a chronic PDAPP mouse study at doses which affected AD pathology in the absence of any toxicity [76].

**Table 2:** Histology scores of mice following 7 days treatment

Treatment (mg/kg/dose)	Duodenum		Ileum	
		
	# animals affected	Mean severity score	# animals affected	Mean severity score
Vehicle	0	NA	0	NA
5 mg/kg LY411575	4*	2	5	3
15 mg/kg ELN475516	0	NA	0	NA
30 mg/kg ELN475516	0	NA	4	0.5
100 mg/kg ELN475516	0	NA	2	1.25
300 mg/kg ELN475516	0	NA	0	NA
600 mg/kg ELN475516	0	NA	2	0.5
1,000 mg/kg ELN475516	0	NA	1	0.5

Small intestine histopathology of mice treated twice daily with the compound and doses indicated in column 1. Duodenum and ileum segments from treated mice were analyzed for cellular homeostasis by histology as indicated in methods. The number of animals affected is indicated next to the dose, as well as the mean severity score for goblet cell hyperplasia in the affected animals. Severity score rating: 0 = no significant change, 0.5 = questionable, 1 = minimal, 2 = mild, 3 = moderate, 4 = severe (*tissue lost from one animal). NA indicates not applicable.

## Discussion

To discover novel APP-selective inhibitors of gamma-secretase, we developed cell-free assays employing recombinant APP or Notch substrates incubated with an enriched enzyme preparation derived from IMR-32 neuroblastoma cells. We utilized this biochemical assay for APP cleavage by gamma-secretase in a high-throughput screen of a chemical library and discovered amino-caprolactam sulfonamide leads, which demonstrated APP-selective inhibition of gamma-secretase activity *in vitro*. The selectivity of our lead series was confirmed in a cellular assay. Comparison of APP selectivity of non-selective reference GSIs in cell-free compared with cellular (SNC) assay reveals a basal 15-22X right-ward shift for selectivity in the cellular assay compared with the cell-free assay with reference non-selective GSI's L-685,458 and LY411575. This observed shift in selectivity between a cell-free assay which measures inhibition of substrate cleavage, versus downstream signaling events, likely reflects a difference between inhibition of Notch cleavage vs. Notch signaling. Our observation is consistent with findings that Notch signaling was only reduced 35 to 48% in cells treated with concentrations of peptidomimetic GSIs which abolished NICD formation to below detectable levels [77].

The benzene caprolactam sulfonamide ELN381463 [59] lowered brain Aβ_1-X _in a concentration dependent manner in PDAPP mice; however, reduction of Aβ_x-40 _was nominal and did not exhibit a concentration dependent effect in two mouse lines tested (PDAPP and FVB). This discordance in a concentration effect on reduction of brain Aβ species cannot be explained by differences in drug levels in the two mouse lines, nor by assay differences, as discussed above. We are not able to explain the basis for this discrepancy; however, our observation suggests efficacy determination of GSI's in AD transgene models should be corroborated in wild type mice (see also Discussion below).

Lead optimization of the caprolactam sulfonamide enabled discovery of a novel pyrazolylazabicyclo(3.3.1)nonane sulfonamide, ELN475516 (this report, and [60]). ELN475516 demonstrated equipotent *in vivo *activity against Aβ_1-X _in PDAPP transgene as well as Aβ_x-40 _in wild type mice, retaining favorable 123X APP-selective inhibition of gamma-secretase. The potency and cellular selectivity of ELN475516 compares favorably with recently disclosed GSI-953 [48] and BMS-708163 [78,79], which demonstrated 45X and 57X selectivity in our hands, with IC_50 _values for inhibition of Aβ production in the SNC assay of 8.0 nM, and 1.13 nM, respectively. The novel sulfonamide inhibitors we describe exhibit all the signatures associated classical gamma-secretase inhibitors: elevation of C99 levels and equipotent inhibition of Aβ40 and Aβ42 production without affecting secreted APP levels. These properties are consistent with those reported for a novel thiophene sulfonamide gamma-secretase inhibitor, GSI-953 [48]. In binding site studies, both ELN318463 and ELN475516 were not able to displace a biotin-labeled active site binding probe in cellular extracts over a range of concentrations tested, suggesting the inhibitors bind gamma-secretase at a site distinct from the active site, consistent with effects reported for GSI-953 [48], as well as non-transition state-based gamma-secretase inhibitors [80]. Interestingly, in the presence of exogenous APP substrate added to the extracts, we were able to detect displacement of the active site isostere at concentrations above the IC_50 _for Aβ inhibition by the compound. This novel observation suggests that occupancy of the substrate binding site on gamma-secretase by APP induces a conformational change bringing the sulfonamide binding site and isostere binding (that is, catalytic) sites in close proximity, enabling displacement of isostere by sulfonamide at supra-physiological concentrations. Although we did not test the effect of other substrates in the competitive displacement assay, it will be interesting to compare the effect, for example, of Notch substrate on displacement of active site isostere by test GSI's in future studies.

The improved selectivity of ELN475516 was tested in a mouse seven-day repeat dose model. Relative to LY411575, a reference non-selective gamma-secretase inhibitor, ELN475516 demonstrated significant reduction of brain Aβ at the lowest dose tested (15 mg/kg/dose, b.i.d.), with no overt signs of toxicity at the highest dose tested (1,000 mg/kg/dose, b.i.d.) at the end of the study, indicating an *in vivo *selectivity >65 based on dose as well as terminal compound concentration in plasma. For this study, a minimum efficacious dose was defined as that dose effecting >25% reduction at study termination (that is, three hours post last dose). Terminal plasma compound levels at the highest dose tested were 12.3 μM, a 17-fold multiple over the cellular IC_50 _for inhibition of Notch signaling. The utility of the mouse as a model for screening of lead gamma-secretase inhibitor candidates against Notch endpoints has been previously described [41]. Our studies employed the wild-type FVB strain of mice for Aβ and Notch endpoints instead of transgenic AD mice as described by Hyde *et al*. to avoid potential transgene model specific influences on *in vivo *inhibitor potency against Aβ endpoints. For example, substrate expression levels [81], as well as PS1 FAD mutations [59,82-84] have been demonstrated to effect a right-ward shift on *in vitro *potencies of gamma-secretase inhibitors. If substrate levels, or PS1 isoform contributes to *in vivo *potency shifts, the effect could potentially confound estimates of *in vivo *selectivity in an APP and/or PS1FAD transgene mouse model. Consistent with the influence of APP expression levels on inhibitor potency, we observed a 33% reduction of brain Aβ_x-40 _in wt FVB mice at three hours post treatment with 30 mg/kg GSI-953, compared with 5 mg/kg producing a similar magnitude effect in Tg2567 mice [48], although it is possible the observed discrepancy may be accounted for by differences in drug exposures in the target organ in FVB mice and Tg2576, or different species of Aβ assayed in the different mouse models queried.

It is noteworthy that all substrate selective GSIs described to date are based on sulfonamide pharmacophore ([47,79,85-87], and this report). Indeed, a first generation aryl sulfonamide GSI, BMS299897 [10,11,88], was demonstrated to avoid Notch inhibition endpoints in the gastro-intestinal tract in a preclinical safety model [28]. Additional arylsulfonamide analogs related to BMS299897 [89], as well as aminocaprolactam sulfonamides related to ELN318463 have been described [79,90]. The APP selectivity of the sulfonamide core appears to be very sensitive to structural modifications. For example, potent and *in vivo *active (bicycle(4:2:1) nonane sulfamide GSI's [91,92], as well as 4-cyclohexyl sulfones [12,93] have been reported as equipotent on APP and Notch. Interestingly, despite equipotent inhibition of APP and Notch processing by gamma-secretase, sulfones are reported to be well tolerated at 3 mg/kg dose in a three- month treatment study in the Tg2576 mouse model [14], although higher doses were not reported. In a similar vein, whereas potent piperdine containing sulfonamide GSI have also been described [90,94-97], APP selectivity has only been reported following incorporation of a pyrazole substituent into the piperdine sulfonamide core [98].

The molecular basis of sulfonamide based GSIs APP selectivity remains to be fully elucidated. We have reported that one contributor of selectivity may be the more potent inhibition of PS1 gamma-secretase compared with PS2 gamma-secretase [59] by sulfonamides. Consistent with this finding, ELN475516 is a five-fold more selective PS1 gamma-secretase inhibitor relative to PS2 gamma-secretase (unpublished). Site directed mutagenesis studies have identified select residues in PS1 which affect AICD processing from APP without affecting NICD production from Notch [99,100], supporting the potential for modulating cleavage by this enzyme in a selective manner. These observations, combined with substituted cysteine accessibility mutagenesis to more precisely map inhibitor binding residues [101,102], offer an avenue to further characterize the biochemical basis of APP selectivity. In summary, the *in vivo *selectivity of ELN475516 from a mouse seven-day safety model corroborates the improved selectivity estimated for this compound in the cellular SNC assay, confirming APP selective inhibition of gamma-secretase *in vivo *by this novel pyrazolylazabicyclo(3.3.1)nonane sulfonamide. ELN475156 represents a validated foundation for further lead optimization to discover APP selective second generation GSIs with improved safety and drug like properties suitable for chronic AD therapy [60,98,103,104].

## Conclusions

Our results showing discordance in reduction of brain Aβ between PDAPP and wild-type FVB mice following treatment with ELN318463 highlight the importance of evaluating GSI's for potency and selectivity in non-transgene models of Alzheimer's disease. The *in vitro *and *in vivo *selectivity of ELN475516 demonstrates that discovery of APP selective GSI's is feasible, and that APP selective GSI's offer potentially safer candidates as therapeutics for Alzheimer's disease.

## Abbreviations

Aβ: amyloid beta-peptide; AD: Alzheimer's disease; APP: amyloid precursor protein; APPsw: Swedish familial Alzheimer's disease mutant isoform of APP: BACE: beta-APP cleaving enzyme; BMS299897: 2-((1*R*)-1-(((4-chlorophenyl)sulfonyl)(2,5-difluorophenyl)amino)ethyl)-5-fluorobenzenebutanoic acid; CHES: 2-(N-Cyclohexylamino)ethanesulfonic acid; CHO: Chinese hamster ovary; CSF: cerebrospinal fluid; CTF: carboxy-terminal fragment; DTT: dithiothreitol; DMSO: dimethyl-sulfoxide; ELISA: enzyme like immunoadsorbent assay; ELN158162: 4-Chloro-N-(3-methoxy-4-(2-(4-methyl-1,3-thiazol-5-yl)ethoxy)benzyl)-*N*-((3*R*)-2-oxoazepan-3-yl)benzenesulfonamide; ELN-318463: *N*-(4-Bromobenzyl)-4-chloro-*N*-((3*R*)-2-oxoazepan-3-yl)benzenesulfonamide; ELN-475516: 10-((4-Chlorophenyl)sulfonyl)-4,5,6,7,8,9-hexahydro-1*H*-4,8-epiminocycloocta(c)pyrazole; FAD: familial Alzheimer's disease; GSI: gamma-secretase inhibitor; HA: hemagglutnin antigen; H&E, hematoxylin and eosin; HEK, human embryonic kidney; Hes, hairy enhancer-of-split; HTS: high through-put screen; KO: knock-out,; L-685,458: *tert*-Butyl (2S,3R,5R)-6-((*S*)-1-((*S*)-1-amino-1-oxo-3-phenylpropan-2-ylamino)-4-methyl-1-oxopentan-2-ylamino)-5-benzyl-3-hydroxy-6-oxo-1-phenylhexan-2-ylcarbamate; LC: liquid chromatography; mAb: monoclonal antibody; LY 411575, (*S*)-2-((*S*)-2-(3,5-Difluorophenyl)-2-hydroxyacetamido)-*N*-((*S*)-5-methyl-6-oxo-6,7-dihydro-5*H*-dibenzo(b,d)azepin-7-yl)propanamide; MES, 2(N-Morpholino)ethanesulfonic acid; Math: mouse atonal homolog; MS: mass spectrometry; Nct: Nicastrin; NICD: Notch Intracellular domain; PAGE: polyacrylamide gel electrophoresis; PAS: periodic acid-Schiff stain; PDAPP: platelet derived growth factor promoter driven AD transgene mouse; sAPP: secreted APP; sAPPβ: secreted APP product of BACE cleavage; SNC: a stable CHO cell line co-expressing APPSw, rat NotchΔE, and an NICD responsive CBF-Luciferase reporter gene; PS: presenilin; RT-PCR: reverse-transcriptase polymerase chain reaction; WGA: wheat-germ agglutnin.

## Competing interests

All authors of this manuscript either were, or currently still are, employees of Elan Pharmaceuticals, Inc. at the time the studies reported in this manuscript were conducted, hold (or held) stock in Elan, and are inventors on patent filings resulting from the work described herein.

## Authors' contributions

GSB, EFB, JB, MSD, JH, SH, LHL, MNM, LM, MLN, KQ, PS, JAT, AWG, JMS, AWK, GS, MAP, SBF, and DS participated in research design. SH , AL, DLA, X-HC, MSD, TE, EG, KH, TH, JJJ, PSK, DK, RLH, ML, JM, MNM, SM, JLM, RM, MLN, HN, LN, LR, CMS, JS, FS, BS, KT, PT, XMY, MY, JW and YZ conducted experiments. GSB, SH, EFB, AL, DLA, JB, RB, MB, X-HC, MSD, EG, JH, KH, TH, JJJ, PSK, LHL, ML, MNM, JLM, RM, LM, MLN, LN, KQ, CMS, PS, BS, JAT, XMY, YZ, AWG, JMS, AWK, DS and GS performed data analysis. GSB, SH, EFB, MB, JH, LHL, AWG, DN and DS wrote, or contributed to writing of manuscript.

## Supplementary Material

Additional File 1 **Figure S1. *In vitro *enzyme kinetics of gamma-secretase preparation on APP and Notch substrates**. The results show that the *in vitro *assay displays Michaelis-Menten kinetics for substrate. Click here for file

Additional File 2 **Figure S2. Neo-epitope specificity of Notch antibody recognizing Notch Intracellular domain**. Click here for file

Additional File 3 **Figure S3. Equipotent inhibition of Aβ40 and Aβ42 by gamma-secretase inhibitors in a cellular assay**. Click here for file

Additional File 4 **Figure S4. Flow-chart of displacement assay with active-site binding affinity ligand in the presence or absence of added APP substrate plus competing sulfonamide**. Click here for file

Additional File 5 **Figure S5. Evolution of sulfonamide structure activity relationship from caprolactam sulfonamides to fused bi-cyclic sulfonamides**. Click here for file
