# Supplementary figures and images for: Amyloid precursor protein selective gamma-secretase inhibitors for treatment of Alzheimer's disease
Source: Alzheimers Res Ther. 2010 Dec 29;2(6):36. doi: 10.1186/alzrt60 (PMC3031881; doi:10.1186/alzrt60)

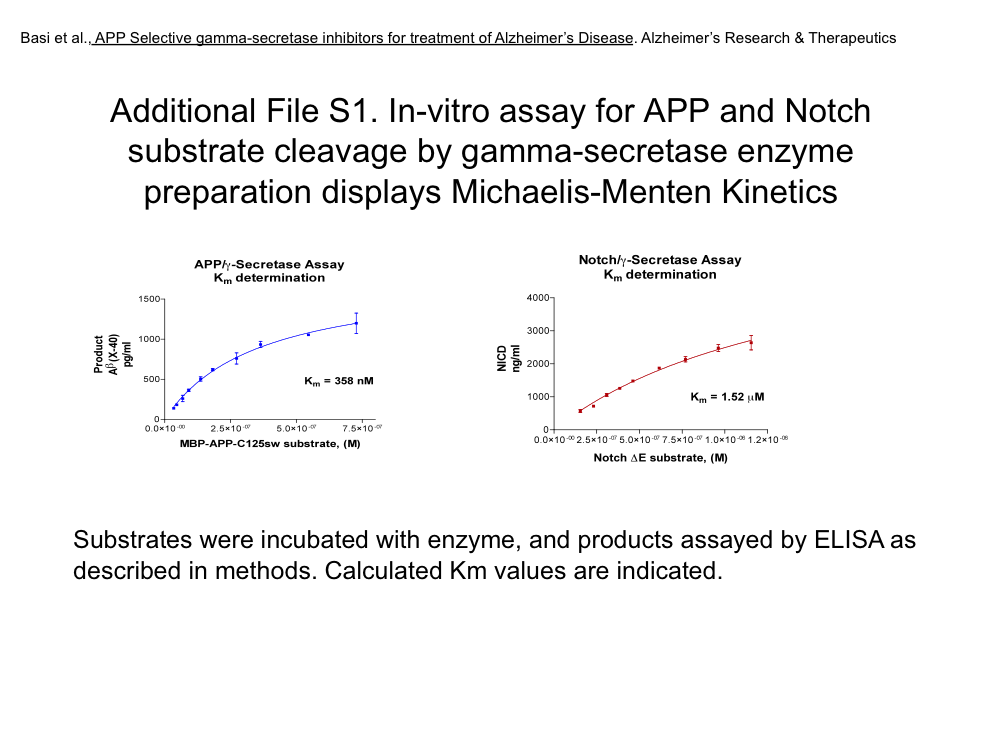

Supplement: Additional File 1 — Figure S1. In vitro enzyme kinetics of gamma-secretase preparation on APP and Notch substrates. The results show that the in vitro assay displays Michaelis-Menten kinetics for substrate. [file alzrt60-S1.TIFF]

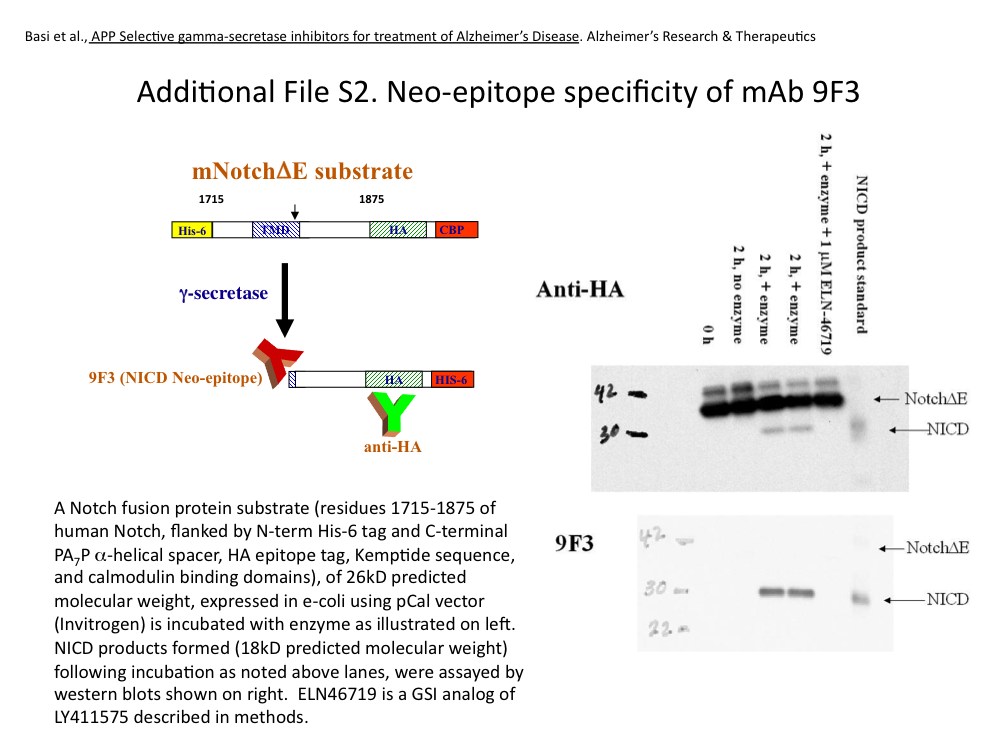

Supplement: Additional File 2 — Figure S2. Neo-epitope specificity of Notch antibody recognizing Notch Intracellular domain. [file alzrt60-S2.TIFF]

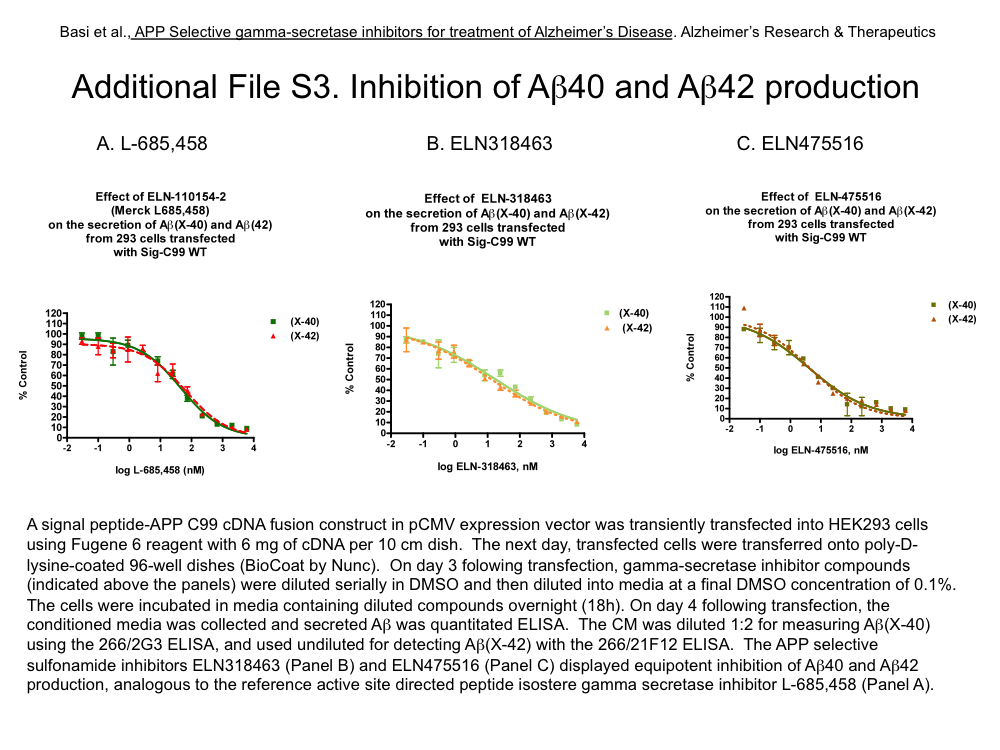

Supplement: Additional File 3 — Figure S3. Equipotent inhibition of Aβ40 and Aβ42 by gamma-secretase inhibitors in a cellular assay. [file alzrt60-S3.TIFF]

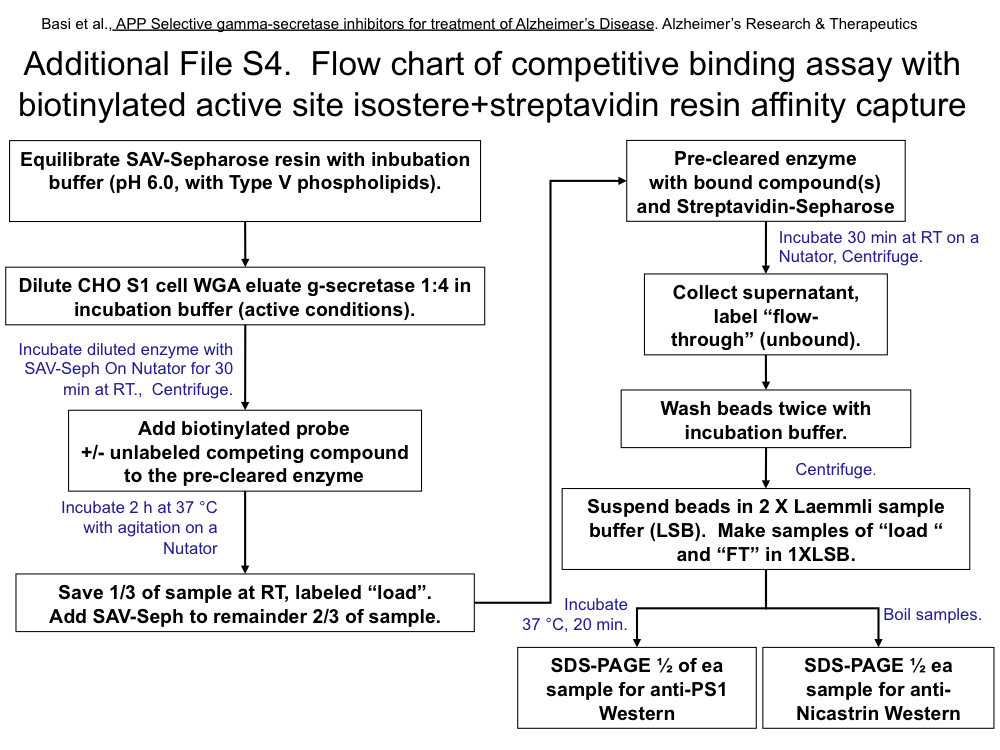

Supplement: Additional File 4 — Figure S4. Flow-chart of displacement assay with active-site binding affinity ligand in the presence or absence of added APP substrate plus competing sulfonamide. [file alzrt60-S4.TIFF]

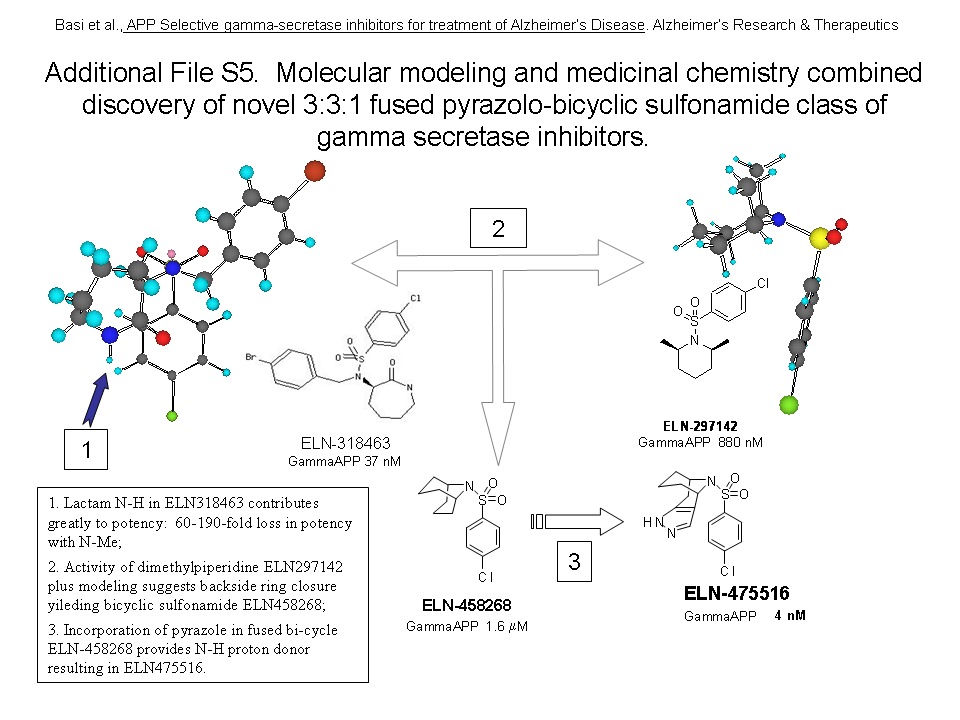

Supplement: Additional File 5 — Figure S5. Evolution of sulfonamide structure activity relationship from caprolactam sulfonamides to fused bi-cyclic sulfonamides. [file alzrt60-S5.TIFF]
